# A Lecture on Hæmaturia or Voiding of Bloody Urine

**Published:** 1839-04-13

**Authors:** N. Chapman

**Affiliations:** Professor of the Theory and Practice of Physic in the University of Pennsylvania


MEDICAL EXAMINER.

DEVOTED TO MEDICINE, SURGERY, AND THE COLLATERAL SCIENCES.

No. 15.] PHILADELPHIA, SATURDAY, APRIL 13, 1839. [Vol. II.

### A LECTURE ON HÆMATURIA OR VOIDING OF BLOODY URINE. *By* N. Chapman, M. D., *Professor of the Theory and Practice of Physic in the University of Pennsylvania.*

[Reported for this Journal.]

These are wretched appellations, not at all conveying a just notion of the affection, which is really a hæmorrhage that may arise from the kidney, the ureter, bladder, or urethra, and should be designated, accordingly, hæmorrhagium renum, hæmorrhagia vesicæ, &c. From its derivation, hæmaturia strictly means mixture of blood, though the English phrase I have given is commonly adopted as its equivalent. The term is differently applied by the authorities, some using it to denote a bleeding from the kidney only, calling that of the bladder cystirrhagia, while others embrace under it the sanguineous effusions of the whole of the urinary apparatus, and in this extended sense, it is now mostly employed, and for the present will be retained by me.

Coming from different sources, and occasioned by a diversity of causes, the symptoms of this hæmorrhage must necessarily be exceedingly modified. Generally, however, it is characterized by obtuse pain, and sense of weight in the loins, sometimes extending down the thigh, with, perhaps, retraction of the testicle, the urine at first high coloured only, then of a darkish red, owing to the dissolved blood, or tolerably clear with small clots floating in it, or the discharge may consist of pure blood, black and grumous, or be entirely suppressed, from the urethra being choked up by coagula. This occurring, the bladder sometimes fills to a painful distension. Distinct, however, from such a condition, there may be great uneasiness in that viscus, dull, heavy, or burning, or pricking sensations, and those of swelling in the perineum, with frequent erections of the penis, and spasm, resembling chordee, attended by tenesmus and strangury, or micturition.

Concomitantly with these local affections, some constitutional disturbance generally exists, evinced by fever, or at least an irregular circulation, and especially by headache, and nausea, and retchings. The symptoms, however, as I have said, are extremely diversified. On some occasions, there is apparently no affection whatever, except the discharge of blood, which passes away without any suffering.

Like every other hæmorrhage, this may proceed from general or local causes, though of the former, instances are of rare occurrence. But it has been seen by myself, and by others, occasionally to appear, consequent on a plethoric condition, with undue determinations of blood to the parts. No doubt whatever can be entertained of its connection with both inflammatory and congestive fevers, as well as other acute affections, in which some portion of the urinary organs has become involved. Certain articles, too, operating through the system, though from a sort of specific affinity, concentrating their force more immediately on the source of the hæmorrhage, lead to its production,—among which are phosphorus and cantharides, and in regard to the latter, whether applied to the skin as a vesicatory, or taken internally.

As a further illustration of the sympathetic production of hæmaturia, it may be mentioned, that it has been noticed as an incident of painful dentition in children, and also from the irritation of worms in the alimentary canal. More frequently, however, it is met with from metastasis of the catamenial or hæmorrhoidal flux, especially or vicariously to these discharges.

Nevertheless, hæmaturia is mostly to be traced to causes operating directly on the part, some violence done to the kidney by blows or falls, or from lifting heavy weights, or leaping, or hard riding. By the irritation of a calculus in the kidney or the ureter, or bladder, it is also induced, and there is a case recorded of its having been brought on by a worm in the second of these positions. Besides which, it is the effect of many organic lesions of these structures, arising from the numerous diseases to which they are exposed.

Every period of life is liable to this hæmorrhage, from childhood to extreme age, though, I think, it is more frequently seen among the old and infirm, broken down by gout, or intemperate or debauched habits.

It may happen where the amount of blood is small, and intimately commingled, or it has undergone some change in its consitution thereby rendered more pallid, or the urine becomes of a more reddish or darker hue from an excess of its saline ingredients, or of lithic acid, a case is presented of some difficulty of discrimination on a superficial examination. But here, we are supplied with a test of great certainty. Dipping a piece of linen into the fluid, however minute the quantity of blood, it is stained of a reddish tinge, which does not take place otherwise.

Not so easy is it, at all times, to determine the precise part of the urinary apparatus whence the blood escapes. As a general rule, however, it will be found in the renal and uretral cases, that, with much more lumbar affection, the blood is usually intimately mixed with the urine, so as to give to it one uniform red appearance, whereas, in the vesical hæmorrhage, it comes away in clots, or flocculi, floating in the urine, and is accompanied by pain, a sense of fulness and tenderness of the pubic region, and those other indications of an affection of the bladder. No perplexity can exist with regard to that of the urethra, for independently of the absence of the symptoms appertaining to the other cases, the blood is emitted without mixture with urine, or an effort to its evacuation. It were fortunate could we come to any satisfactory conclusion concerning the several pathological conditions giving rise to this hæmorrhage. But sometimes it cannot be done, though much in other instances may be accomplished by a diligent investigation of the history of the case, and comparison of symptoms, as well as by a careful inspection of the urine, which containing mucus pus or gravelly deposits, considerable light is shed on the subject.

Excepting in those acute cases, in a sound and vigorous constitution, owing to a general redundancy of blood, or local accumulations of it, or its occurring metastatically or vicariously, and here, if not salutary it does little or no injury, hæmaturia is to be deprecated, not so much from any danger in itself, as its indicating some serious derangements of the organs, whence the hæmorrhage proceeds. Nevertheless, it may be immediately alarming in appearance, from an immense loss of blood.

In the winter of 1831, I attended an elderly gentleman, of a very full, plethoric habit, who voided on an average about three quarts of nearly pure blood in the twenty-four hours for three successive returns of this period, and which continued in a diminished quantity till he became nearly exhausted.

Not many months afterwards, he had a repetition of the attack, in which he lost, in the aggregate, perhaps, an equal amount of blood, though not in so short a time. Many recurrences of it had he subsequently, on each occasion profusely, till he died, which was not, however, of the hæmorrhage. Death indeed from it very seldom happens. The register of the Vienna Hospital shows only a solitary instance, out of thirteen thousand six hundred and forty-seven cases of the affection.

Effusions from the kidneys and bladder are of course the most copious. Being, however, of a vital character, they either spontaneously cease, or are readily checked, and seldom prove detrimental. But it is very different with the physical, or those dependent on organic derangements. The cause here enduring permanently, so must the effect, or if the latter be removed, it is temporarily, reverting again at no distant interval. The most ominous occurrence, however, of hæmaturia, is in the advanced stages of low fevers, and other diseases of extreme exhaustion. But this happening it is purely passive, a mere leakage of blood, from the impairment or absolute extinguishment of vital power, and can scarcely be deemed a genuine hæmorrhage. On the whole, our prognosis must be derived, from the estimate formed of the condition of the parts, and the system generally, with which the hæmorrhage may be associated.

Of the autopsic phenomena in the acute and vital form of hæmaturia, I have no precise information. They may, however, be conjectured from the history of the disease, varied by the seat and cause of it. The kidney, itself, probably presents similar appearances to those in other parenchematous hæmorrhages, and the ureters, bladder, and urethra, such as are observed in the mucous membranes generally. But, in chronic cases, every variety of disorganization of structure of the kidney, and bladder especially, has been reported.

Equally may its pathology be deduced from analogy, and I shall, therefore, occupy little time on a point, the discussion of which were merely a recapitulation of what has been, on preceding occasions, amply expounded. Excepting the cases caused by acts of violence, or organic changes, every other may be referred to the mode in which I have shown vital hæmorrhage to take place. Even the former are not invariably otherwise induced. Mostly, where the lesion is excessive, a rupture of vessels is to be presumed, though these may escape,—and certainly where the injury is less, it operates merely to the irritation of the capillaries through which the blood is effused.

In the treatment of this, we are to be guided by the same general principles as in other hæmorrhages, having special regard to the existing pathological condition. The case exhibiting local phlogosis, or active congestion, or febrile excitement, we resort to venesection, cupping or leeching over the lumbar region, slight purging, demulcents, and especially an infusion of peach leaves, or of the petals of the red rose. The two latter articles, though apparently very simple, are the most efficacious with which I am conversant.

Many instances I have seen, that resisting more energetic means, were relieved by these mild remedies. It is customary, I am aware, to rely here mainly on those astringents which are supposed so efficacious in the kindred affections. But, really, I have witnessed no decisive advantage from them, and, certainly, they are more appropriate to another state of the hæmorrhage, presently to be noticed. As usual is it to administer largely the diuretics, the nitrate of potash particularly, with the beverages promotive of its operation, to which I entirely object, provided the kidney be the seat of the effusion, and already unduly excited. The reverse, indeed, is the indication, under such circumstances, rather to allay, than provoke any increase of action, by forcing it to greater secretory efforts.

In a case of less activity, if admissible at all, venesection must be comparatively moderate, and topical bleeding chiefly used. Blistering over the lumbar region is entitled to great confidence. That it has been strenuously opposed, is not unknown to me, though on false grounds. From experience I have learnt, that it may be as safely and efficaciously adopted, as in any of its ordinary employments. But the blister should be permitted to remain on long enough only to produce simply a rubescence of the skin, by which its beneficial effect is amply attained, and the danger of strangury prevented.

This is the conjuncture to which I alluded, when the acetate of lead, the sulphate of alumine, the muriated tincture of iron, the gallic acid, the elixir of vitriol, the creosote, and alike articles, may be tried with a fairer prospect of success. Yet I confess, that I have found them, even here, of very equivocal utility. As to the uva ursi, so commended by some, it has totally disappointed my expectations. But the tincture, in combination with gallic acid, is, of late, very favourably spoken of, which I have not used. The best article known to me is the spirit of turpentine.

Emetics had formerly great reputation, and I believe deservedly, though I have never had recourse to them,—finding, on all occasions to which I deemed them appropriate, measures less disagreeable to answer.

Much of this treatment is equally suited to hæmorrhage of the bladder. The topical applications are usually made to the pubes or the sacrum, as nearer the seat of the affection. Yet, leeches to the groins and perineum are more effectual. It has also been proposed to inject into the bladder cold mucilages, or astringent fluids, according to the indication.

Great suffering, I have said, is sometimes felt by a retention of blood from an occlusion of the urethra. This is removed, at once, by the introduction of a bougie or catheter.

It is obvious, that the management of these cases requires to be further accommodated to their peculiarities. Excited, for instance, by calculi, and especially when a small one is lodged in the ureter, though the hæmorrhage may be slight, the agony is extreme. Disregarding the effusion, our attention must be directed to the mitigation of pain, and to the passage of the calculus through the tube, by which complete relief is only afforded. Fortunately, the means are the same with both intentions, consisting of general and local bleeding to a great extent, the warm bath, and opiates, chiefly as enemata. Caused by any material structural lesions, it is better, in an inflammatory condition of the parts, to allow the hæmorrhage to continue, as serviceable, unless it be inordinately profuse. Then, or in a case of original debility, the balsams or terebenthinates are to be preferred, in reference to a suppression of the effusion, and with a view to the alleviation of the pain, commonly an attendant, the opiates.

In the arrestation of this hæmorrhage, when active, from whatever source it may emanate, or the cause occasioning it, much will depend on the adoption of a course of living, to the total exclusion of every article, whether of food or drink, of a heating or stimulating kind, and, scarcely less, on the strictest observance of a state of rest during and for some time after the effusion. Equally does the latter clause of this precept apply to the opposite state of the affection,—but the diet should be more nutritious, though still bland, or without any irritating qualities.

Need it be added, that before dismissing the case, the pathological condition to which the effusion is owing, is carefully to be ascertained, with a view to its rectification or entire removal?

